# Joint and Independent Associations of Gestational Weight Gain and Pre-Pregnancy Body Mass Index with Outcomes of Pregnancy in Chinese Women: A Retrospective Cohort Study

**DOI:** 10.1371/journal.pone.0136850

**Published:** 2015-08-27

**Authors:** Chunming Li, Yajun Liu, Weiyuan Zhang

**Affiliations:** 1 Department of Obstetrics, Women's Hospital, School of Medicine, Zhejiang University, Hangzhou, Zhejiang Province, China; 2 Department of Obstetrics, Beijing Obstetrics and Gynecology Hospital, Capital Medical University, Beijing, China; Indiana University School of Medicine, UNITED STATES

## Abstract

**Objective:**

To explore the joint and independent effects of gestational weight gain (GWG) and pre-pregnancy body mass index (BMI) on pregnancy outcomes in a population of Chinese Han women and to evaluate pregnant women’s adherence to the 2009 Institute of Medicine (IOM) gestational weight gain guidelines.

**Methods:**

This was a multicenter, retrospective cohort study of 48,867 primiparous women from mainland China who had a full-term singleton birth between January 1, 2011 and December 30, 2011. The independent associations of pre-pregnancy BMI, GWG and categories of combined pre-pregnancy BMI and GWG with outcomes of interest were examined using an adjusted multivariate regression model. In addition, women with pre-pregnancy hypertension were excluded from the analysis of the relationship between GWG and delivery of small-for-gestational-age (SGA) infants, and women with gestational diabetes (GDM) were excluded from the analysis of the relationship between GWG and delivery of large-for-gestational-age (LGA) infants.

**Results:**

Only 36.8% of the women had a weight gain that was within the recommended range; 25% and 38.2% had weight gains that were below and above the recommended range, respectively. The contribution of GWG to the risk of adverse maternal and fetal outcomes was modest. Women with excessive GWG had an increased likelihood of gestational hypertension (adjusted OR 2.55; 95% CI = 1.92–2.80), postpartum hemorrhage (adjusted OR 1.30; 95% CI = 1.17–1.45), cesarean section (adjusted OR 1.31; 95% CI = 1.18–1.36) and delivery of an LGA infant (adjusted OR 2.1; 95% CI = 1.76–2.26) compared with women with normal weight gain. Conversely, the incidence of GDM (adjusted OR 1.64; 95% CI = 1.20–1.85) and SGA infants (adjusted OR 1.51; 95% CI = 1.32–1.72) was increased in the group of women with inadequate GWG. Moreover, in the obese women, excessive GWG was associated with an apparent increased risk of delivering an LGA infant. In the women who were underweight, poor weight gain was associated with an increased likelihood of delivering an SGA infant. After excluding the mothers with GDM or gestational hypertension, the ORs for delivery of LGA and SGA infants decreased for women with high GWG and increased for women with low GWG.

**Conclusions:**

GWG above the recommended range is common in this population and is associated with multiple unfavorable outcomes independent of pre-pregnancy BMI. Obese women may benefit from avoiding weight gain above the range recommended by the 2009 IOM. Underweight women should avoid low GWG to prevent delivering an SGA infant. Pregnant women should therefore be monitored to comply with the IOM recommendations and should have a balanced weight gain that is within a range based on their pre-pregnancy BMI.

## Introduction

Obesity has been designated as one of the most important global health threats worldwide, and its prevalence has been increasing among women of reproductive age. Pregnant women constitute an important subpopulation with an elevated risk of obesity due to excessive weight gain. It has been shown that maternal obesity and excessive gestational weight gain (GWG) are associated with adverse obstetric and neonatal outcomes [1– 2]. Moreover, becoming pregnant while overweight or obese or gaining too much weight during pregnancy is associated with the added burden of chronic disease, which deserves particular attention. However, understanding these associations is complex because both body mass index (BMI) and GWG are closely related to lifestyle, exercise and genetic traits.

To support optimal pregnancy outcomes, the World Health Organization (WHO) recommended that the Institute of Medicine (IOM) develop guidelines for weight gain during pregnancy [3]. However, ethnic disparity in gestational outcomes was not fully explained in these guidelines, and related studies were mostly conducted on Caucasian populations. Limited data are available regarding whether the results are appropriate for Chinese women. Hence, there is a need to determine the contextual relevance of these recommendations in China and to thoroughly assess the contributions of GWG and pre-pregnancy BMI to pregnancy outcomes.

## Materials and Methods

This study was approved by the Ethics Committees of the individual hospitals involved (see acknowledgments) and conformed to the guidelines of the Helsinki agreement and its amendments. The National Research Ethics Service had previously approved the anonymous use of these data for research purposes; therefore, individual informed consent was not required. The data were analyzed anonymously.

This retrospective, multicenter screening analysis was restricted to healthy Han nulliparous women having singleton and full-term births between January, 1, 2011 and December, 31, 2011 in mainland China (except Hong Kong and Macau). Clinical medical records are routinely updated beginning during the first prenatal visit (within the first 12 weeks of pregnancy) and include general information, disease history, and complications during pregnancy as well as intrapartum and neonatal outcomes. We designed a specific questionnaire and collected information from 39 specialized and general secondary or tertiary hospitals of 14 provinces in the northeastern, northwestern, northern, central, eastern, southern, and southwestern areas of China. Clinical information was collected retrospectively from all of the patients by reviewing their clinical medical records, not by face-to face interactions or direct telephone calls. All of the data were collected by specially trained medical staff and were randomly crosschecked by designated staff to ensure validity. Women with pregnancies that were complicated by multiple gestations, known diabetes or chronic hypertension; women < 18 years old; and women for whom data on pre-pregnancy BMI or GWG were missing were excluded. Finally, a total of 48,867 women were included in this study.

Pre-pregnancy BMI (kg/m^2^) was calculated using the maternal weight and height recorded at the first prenatal visit within the first 12 weeks of pregnancy. GWG was defined as the difference between the maternal weight recorded for each woman at the delivery unit and the maternal weight recorded at the first prenatal visit. All of the women were categorized according to the WHO classification and IOM guidelines as follows: for those with a BMI less than 18.5 kg/m^2^ (underweight), the recommended weight gain was between 12.5 and 18 kg; for those with a BMI between 18.5 and 24.9 kg/m^2^ (normal weight), the recommended weight gain was between 11.5 and 16 kg; for those with a BMI between 25 and 29.9 kg/m^2^ (overweight), the recommended weight gain was between 7 and 11.5 kg; and for those who had a BMI of 30 kg/m^2^ or more (obese), the total recommended weight gain was between 5 and 9 kg (Table 1). Women with a GWG within the IOM-recommended range were categorized as having an adequate GWG. Women with a GWG less than the IOM recommendation were categorized as having an inadequate GWG. Women with a GWG greater than the IOM recommendation were categorized as having an excessive GWG.

**Table 1:** New Recommendations for Total Weight Gain during Pregnancy According to Pre-pregnancy Body Mass Index.

Pre-pregnancy BMI	BMI^a^ (kg/m^2^) (WHO)	Total weight gain range (kg)
**Underweight**	<18.5	12.5–18
**Normal weight**	18.5–24.9	11.5–16
**Overweight**	25.0–29.9	7–11.5
**Obese**	≥30.0	5–9

^a^BMI: body mass index

The main purpose of this study was to survey the impact of GWG on pregnancy outcomes within the population; therefore, data on maternal outcomes (such as pregnancy-induced hypertension, gestational diabetes (GDM), and postpartum hemorrhage), peripartum outcomes (such as cesarean delivery, including elective and emergency cesarean section; assisted vaginal delivery; and vaginal delivery), and neonatal outcomes (such as small for gestational age (SGA) and large for gestational age (LGA)) were collated and analyzed.

Pregnancy-induced hypertension was diagnosed by systolic blood pressure ≥ 140 mmHg or diastolic blood pressure ≥ 90 mmHg that developed after 20 weeks of gestation and returned to normal within 12 weeks postpartum. GDM was diagnosed using the oral glucose tolerance test at 24–28 weeks of pregnancy in accordance with the IADPSG criteria [4]. Postpartum hemorrhage was defined as more than 500 ml of vaginal bleeding within 24 h after birth. Assisted vaginal delivery included forceps delivery, vacuum extraction delivery, assisted breech delivery and breech extraction. LGA and SGA refer to infants whose birth weights were greater than and less than the 90th and 10th percentile, respectively, adjusted for gestational age (sex- and parity-specific), according to a Chinese reference curve. Diagnoses were obtained by utilizing the International Classification of Diseases (ICD-10) codes, according to the hospital medical records.

Maternal age at birth, smoking, alcohol consumption, maternal height, social status defined by education, length of gestation and residential area (urban or rural) were regarded as potential confounding factors and were included as covariates in the adjusted analyses.

## Statistical Analyses

The data were entered into an Epi Info 7 spreadsheet and analyzed using the SPSS software package (version 18.0; SPSS Inc, Chicago, IL, USA). The continuous variables were expressed as the mean ± standard deviation as determined by ANOVA. The chi-square test was used to compare maternal demographic characteristics and pregnancy outcomes between each category and the frequencies of the events. A multiple logistic regression model controlling for potential confounders was used to calculate odds ratios (ORs) and 95% confidence intervals (CIs) for outcomes based on GWG or BMI. The reference categories were normal pre-pregnancy BMI or weight gain within the guidelines, as defined above. The significance of the trends in the BMI- and GWG-specific variables obtained by cross-classifying the BMI group and the GWG group was tested in the same models by assigning an ordinal numeric value to each dummy variable. Normal-weight women with adequate GWG were used as a reference. Statistical significance was established when the *P* value was less than 0.05.

## Results

### Description of the Study Population

The general characteristics of the study population according to maternal pre-pregnancy BMI and GWG category are presented in S1 Table. A detailed description of the characteristics of the mothers based on maternal pre-pregnancy BMI was previously published [5]. Compared with the mothers with adequate GWG, the mothers with excessive GWG were older, taller, had a higher pre-pregnancy BMI, reported a higher education level, and were more likely to live in urban areas. Mothers with inadequate GWG were younger, shorter, reported a lower education level and were more likely to live in rural areas compared with the mothers with adequate GWG. A low percentage of the pregnant women in our survey consumed alcohol and cigarettes.

Of the 48,867 women eligible for this study, 6,424 (13.15%) of the subjects were underweight entering pregnancy, 37,359 (76.45%) were of normal weight, 4,497 (9.2%) were overweight and 587 (1.2%) were obese based on their pre-pregnancy BMI. Compared with the healthy-weight women, a significantly higher proportion of the obese women experienced adverse feto-maternal outcomes (Table 2) [5]. Briefly, the obese women had a higher likelihood of pregnancy-induced hypertension, GDM, postpartum hemorrhage, delivery of an LGA infant and caesarean delivery. Conversely, the underweight group had a higher likelihood of delivering an SGA infant but a lower risk of pregnancy-induced hypertension, GDM, caesarean delivery, and delivery of an LGA infant.

**Table 2:** Relative Risk Estimates of Maternal and Neonatal Outcomes in Relation to BMI^a^.

	Underweight Adjusted RR (95% CI)	Normal Adjusted RR (95% CI)	Overweight Adjusted RR (95% CI)	Obese Adjusted RR (95% CI)
**Gestational hypertension**	0.61[0.51–0.74]**	1	2.91[2.56–3.03]**	8.79[7.06–10.96]**
**Gestational diabetes**	0.73[0.62–0.86]**	1	2.22[1.98–2.49]**	5.24[4.23–6.51]**
**Postpartum hemorrhage**	0.93[0.80–1.08]	1	1.50[1.30–1.74]**	1.55[1.07–2.24]*
**Cesarean delivery**	0.68[0.64–0.71]**	1	1.49[1.40–1.59]**	2.64[2.18–3.19]**
**Elective CS** ^**d**^	0.93[0.85–1.01]	1	1.44[1.31–1.59]**	1.24[1.0–1.55]
**Emergency CS** ^**d**^	1.08[0.99–1.17]	1	0.69[0.63–0.76]**	0.8[0.65–1.07]
**Assisted vaginal delivery**	1.13[0.93–1.37]	1	0.89[0.70–1.12]	0.26[0.08–0.81]*
**Vaginal delivery**	1.49[1.41–1.57]**	1	0.65[0.61–0.70]**	0.39[0.32–0.47]
**SGA** ^**b**^	1.66[1.44–1.91]**	1	0.73[0.59–0.91]**	1.09[0.67–1.77]
**LGA** ^**c**^	0.54[0.47–0.62]**	1	2.55[2.32–2.80]**	3.95[3.20–4.87]**

^a^BMI: body mass index

^b^SGA and ^c^LGA: small for gestational age and large for gestational age, defined as the < 10th percentile and > 90th percentile, respectively, of sex-specific reference curves

^d^CS: cesarean section.

Based on multiple logistic regression models adjusted for maternal age at birth, smoking, alcohol consumption, maternal height, social status defined by education, length of gestation, residential area (urban or rural) and gestational weight gain.

**Significantly different from the estimate of the reference category (p < 0.01)

*significantly different from the estimate of the reference category (p < 0.05).

**Fig 1:**
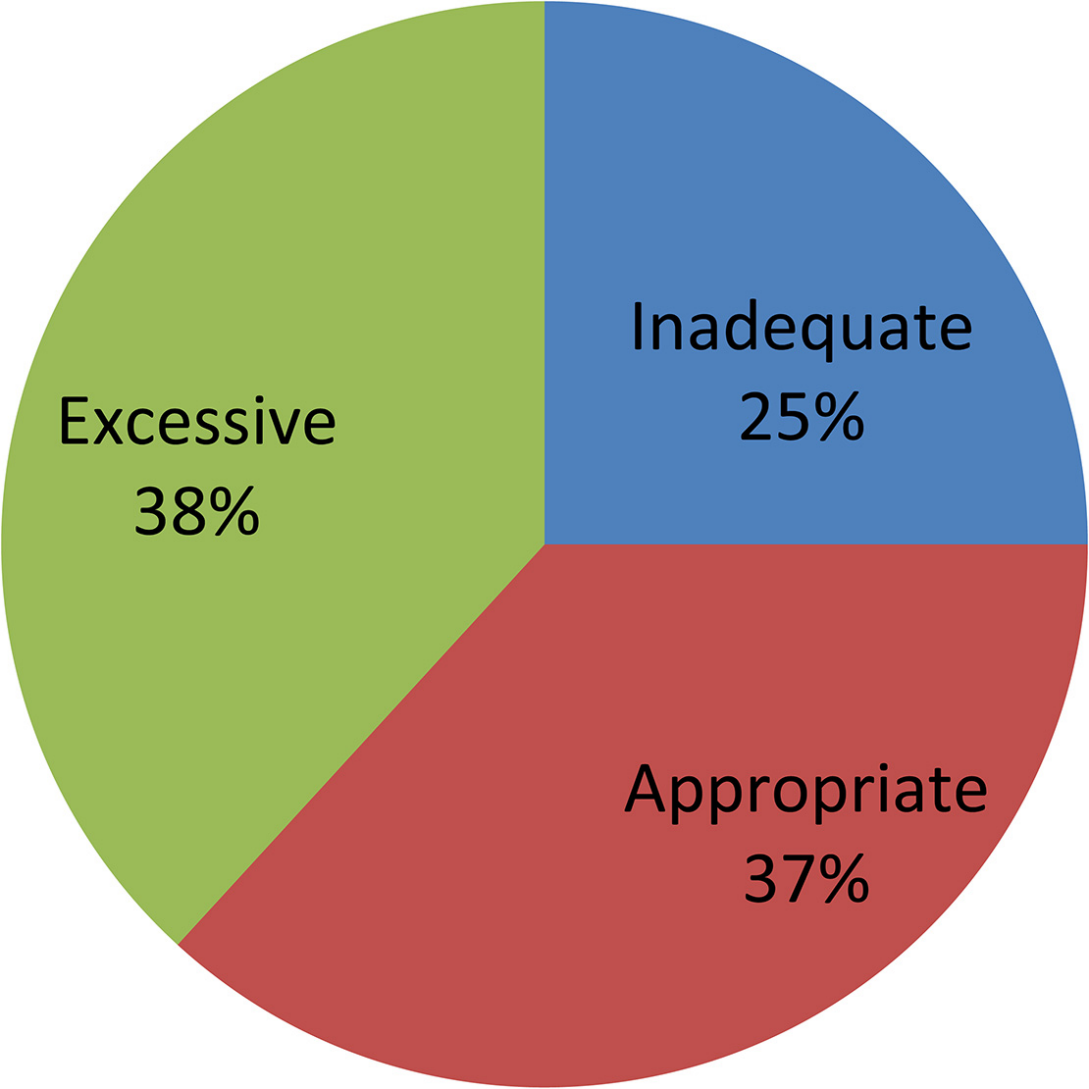
Gestational weight gain in China during 2011.

Maternal weight gain during pregnancy decreased with increasing pre-pregnancy BMI. The mean observed GWG was 14.47 ± 4.98 kg in the whole population, 15.47 ± 4.97 kg in the underweight women, 14.51 ± 4.89 kg in the normal-weight women, 12.95 ± 5.25 kg in the overweight women and 12.69 ± 6.12 kg in the obese women. More than half of the Chinese women did not gain the appropriate amount of weight during their pregnancy; 38.2% of the women gained more than the recommended weight, 36.8% stayed within the guidelines, and 25% gained less than the recommended weight (Fig 1).

**Fig 2:**
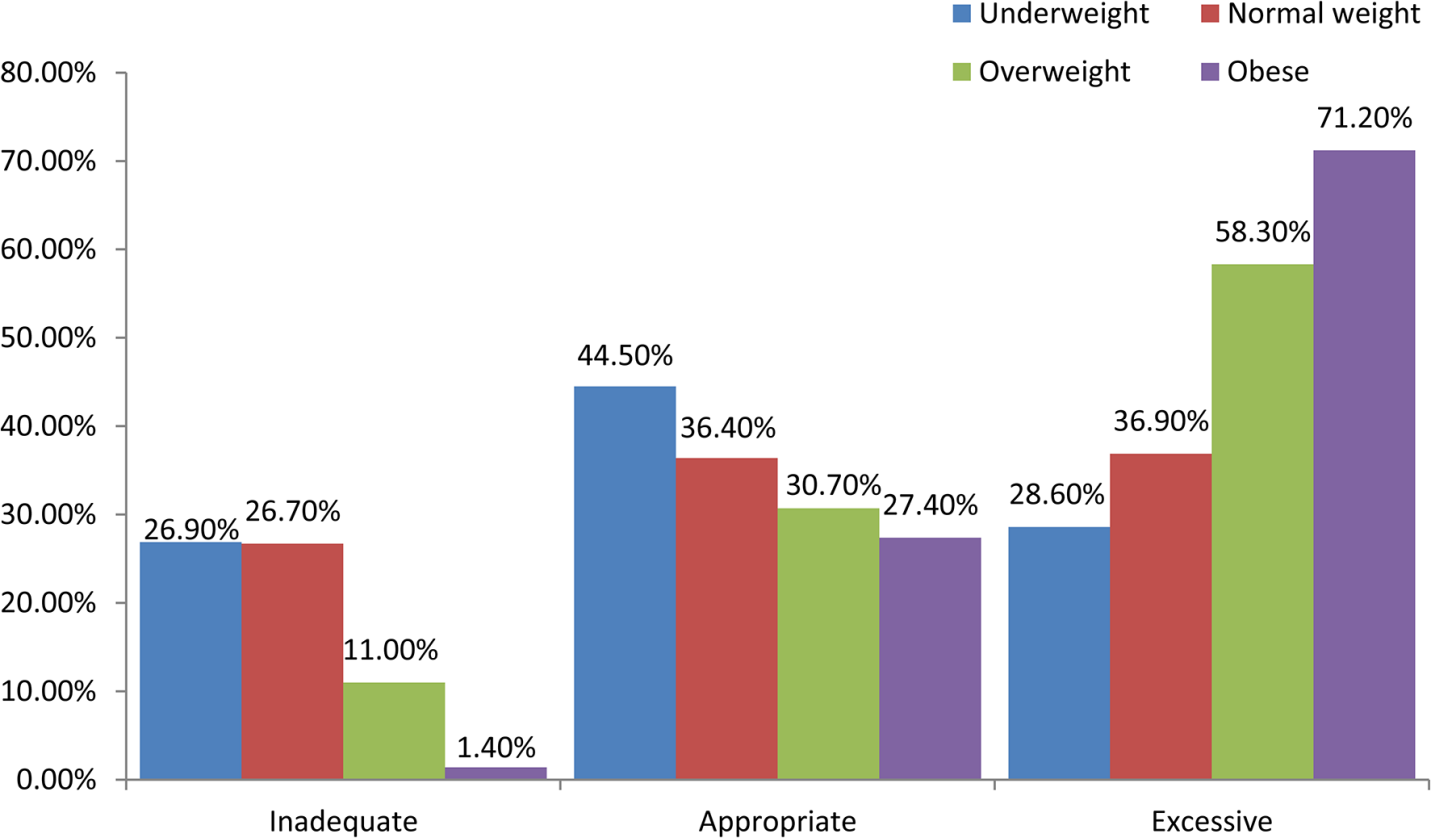
Proportion of pre-pregnancy body size satisfaction status in gestational weight gain groups.

According to the 2009 IOM report, a woman’s pre-pregnancy weight is a primary determinant of how much weight she will gain while pregnant. Fig 2 illustrates the distribution of weight gain according to BMI in our population. Nearly half (44.5%) of the underweight women gained weight within the IOM-recommended range. The majority of the cases of inadequate weight gain occurred among the underweight group (26.9%), whereas inadequate weight gain was rarely observed among the obese women (1.4%). The women who were obese prior to pregnancy were more likely to gain outside of the recommended range during their pregnancy; in our population, up to 71.2% of these women gained weight above the recommended range. Ultimately, the women who did not have a healthy weight prior to pregnancy were more likely to gain outside of the recommended range during their pregnancy.

### GWG and associations with adverse pregnancy outcomes

The number of women with various pregnancy outcomes according to GWG is shown in Table 3. Women with excessive weight gain were more likely to experience pregnancy complications compared to the women with adequate GWG (5.4% vs. 2.7% for pregnancy-induced hypertension, 4.5% vs. 3.5% for postpartum hemorrhage, 56.3% vs. 53.5% for cesarean delivery, 30.8% vs. 26.8% for emergency cesarean delivery and 10.1% vs. 5.6% for delivery of an LGA infant). In contrast, the women who did not reach the optimal weight recommended by the IOM were significantly more likely to experience GDM (5.4% vs. 4.3%) and to give birth to an SGA infant (3.6% vs. 2.4%) compared with those whose weight gain was within the recommended range.

**Table 3:** Pregnancy, Peripartum and Neonatal Outcomes According to Maternal Weight Gain.

Outcome	Inadequate GWG^a^ N = 12223	Adequate GWG N = 17978	Excessive GWG N = 18666	*P* value
**Gestational hypertension**	224 (1.8%)	494 (2.7%)	999 (5.4%)	< 0.01
**Gestational diabetes**	664 (5.4%)	767 (4.3%)	739 (4.0%)	< 0.01
**Postpartum hemorrhage**	316 (2.6%)	621 (3.5%)	842 (4.5%)	< 0.01
**Cesarean delivery**	6370 (52%)	9614 (53.5%)	10505 (56.3%)	< 0.01
**Elective CS** ^**d**^	4757 (74.7%)	7082 (73.7%)	7270 (69.2%)	< 0.01
**Emergency CS** ^**d**^	1613 (25.3%)	2532 (26.3%)	3235 (30.8%)	< 0.01
**Assisted vaginal delivery**	193 (1.6%)	331 (1.8%)	356 (1.9%)	0.09
**Vaginal delivery**	5655 (46.2%)	8033 (44.7%)	7805 (41.8%)	< 0.01
**SGA** ^**b**^	439 (3.6%)	434 (2.4%)	425 (2.3%)	< 0.01
**LGA** ^**c**^	551 (4.5%)	1005 (5.6%)	1889 (10.1%)	< 0.01

^a^GWG: gestational weight gain

^b^SGA and ^c^LGA: small for gestational age and large for gestational age, defined as the < 10th percentile and > 90th percentile, respectively, of sex-specific reference curves.

^d^CS: cesarean section.

Adequate gestational weight gain: underweight, 12.5–18 kg; normal, 11.5–16 kg; overweight, 7–11.5 kg; and obese, 5–9 kg.

Logistic regression was also used to explore the association of the absolute risk of the various pregnancy outcomes with abnormal GWG, controlling for confounders (Table 4). The results showed that excessive weight gain was significantly associated with an increased risk of pregnancy-induced hypertension (OR 2.55; 95% CI = 1.92–2.80). GDM was only strongly associated with low GWG (OR 1.64; 95% CI = 1.20–1.85). The women with excessive GWG had more than a 1.30-fold increased risk of postpartum hemorrhage relative to the women whose weights were within the IOM-recommended range. Compared to the consistently and significantly increased risk of caesarean delivery with increasing BMI, GWG was only modestly associated with this risk. The mothers who gained excess weight during pregnancy were 1.3 (95% CI = 1.18–1.36) times more likely to undergo caesarean delivery compared with the mothers who maintained a healthy weight gain during pregnancy. In the cases in which the woman’s body weight increased above the maximum recommended weight during pregnancy, the frequency of emergency cesarean delivery was increased (OR 1.25; 95% CI = 1.17–1.32). For the women with a weight gain that was lower than the IOM recommendation, there was an increased prevalence of delivering an SGA infant (OR 1.51; 95% CI = 1.32–1.72), and the reverse pattern was observed for the risk of delivering an LGA infant; the women with a weight gain that was above the recommended range were two times more likely to deliver an LGA infant compared with those who gained the recommended amount of weight (OR 2.1; 95% CI = 1.76–2.26).

**Table 4:** Risk of Adverse Pregnancy Outcomes According to the Institute of Medicine Guidelines for GWG^a^ by Adjusted Odds Ratios and 95% Confidence Intervals.

	Inadequate GWG	Excessive GWG
**Gestational diabetes**	1.64[1.20–1.85]**	0.96[0.84–1.26]
**Gestational hypertension**	0.7[0.60–0.82]**	2.55[1.92–2.80]**
**Postpartum hemorrhage**	0.73[0.64–0.84]**	1.30[1.17–1.45]**
**Cesarean delivery**	0.91[0.91–0.99]*	1.31[1.18–1.36]**
**Elective CS** ^**d**^	1.05 [0.98–1.13]	0.80 [0.76–0.85]**
**Emergency CS** ^**d**^	0.95 [0.89–1.02]	1.25 [1.17–1.32]**
**Vaginal delivery**	1.08 [1.03–1.13]**	0.89 [0.85–0.93]**
**Small for gestational age** ^**b**^	1.51[1.32–1.72]**	0.94[0.82–1.08]
**Large for gestational age** ^**c**^	0.80[0.72–0.89]**	2.1[1.76–2.26]**

^a^GWG: gestational weight gain

^b^small for gestational age and ^c^large for gestational age, defined as the < 10th percentile and > 90th percentile, respectively, of sex-specific reference curves;

^d^CS: cesarean section.

Adequate gestational weight gain: underweight, 12.5–18 kg; normal, 11.5–16 kg; overweight, 7–11.5 kg; and obese, 5–9 kg; Adequate gestational weight gain was set as a reference.

Based on multiple logistic regression models adjusted for maternal age at birth, smoking, alcohol consumption, maternal height, social status defined by education, length of gestation, residential area (urban or rural) and pre-pregnancy body mass index.

**Significantly different from the estimate for the reference category (p < 0.01)

*significantly different from the estimate for the reference category (p < 0.05).

### Associations related to inadequate and excessive GWG across BMI groups

Tables 5 and 6 separately present the effects of excessive (Table 5) and inadequate GWG (Table 6) on obstetric and neonatal outcomes in different maternal BMI strata. We demonstrated that the effects of pre-pregnancy BMI and GWG on some of the pregnancy outcomes are independent of each other and that the effects are additive, especially on neonatal weight. For the underweight, normal-weight and overweight women, a GWG < the IOM recommendation increased the likelihood of giving birth to an SGA infant (OR 2.8; 95% CI = 2.4–3.4, OR 1.5; 95% CI = 1.3–1.8, OR 1.4; 95% CI = 1.1–1.9, respectively). A GWG > the IOM recommendation significantly increased the likelihood of delivering an LGA infant in all of the maternal BMI classes, especially the obese women (OR 4.3; 95% CI = 3.5–4.8). Moreover, excessive GWG had a statistically significant effect on cesarean delivery and pregnancy-induced hypertension, and these increases were primarily related to the natural course of weight gain among the overweight and obese women. Indeed, studies of women with normal pre-pregnancy BMIs indicate that women who gain weight beyond the IOM-recommended range have an increased risk of postpartum hemorrhage and delivery of an LGA infant.

**Table 5:** Multivariate Analysis of Outcomes Among Women Whose Weight Gain was Above that Recommended in the Institute of Medicine Guidelines.

Outcome	Underweight OR (95% CI)	Normal Weight OR (95% CI)	Overweight OR (95% CI)	Obese OR (95% CI)
**Gestational hypertension**	2.1[1.4–3.1]*	0.6[0.4–1.0]*	1.5[1.2–2.0]*	2.3[2.1–2.7]*
**Gestational diabetes**	NS^a^	1.0[0.9–1.2]	0.7[0.6–0.8]*	0.6[0.6–0.7]*
**Postpartum hemorrhage**	1.2[0.9–1.7]*	1.5[1.3–1.7]*	1.2[1.1–1.4]*	1.2[1.1–1.4]*
**Cesarean delivery**	1.1[1.1–1.4]	1.0[1.0–1.1]	1.3[1.2–1.4]*	1.9[1.7–2.1]*
**Small for gestational age** ^**b**^	0.9[0.8–1.2]*	0.7[0.6–0.8]	0.6[0.5–0.8]*	0.6[0.5–0.8]*
**Large for gestational age** ^**c**^	1.3[1.1–1.7]*	1.7[1.5–1.9]*	1.8[1.5–1.9]*	4.3[3.5–4.8]*

^a^NS: the number in this category was too small to analyze; small for gestational age^b^ and large for gestational age^c^, defined as the < 10th percentile and > 90th percentile, respectively, of sex-specific reference curves.

Based on multiple logistic regression models adjusted for maternal age at birth, smoking, alcohol consumption, maternal height, social status defined by education, length of gestation, and residential area (urban or rural).

*Significantly different from the estimate for the reference category (p < 0.05).

**Table 6:** Multivariate Analysis of Outcomes Among Women Whose Weight Gain was Below that Recommended in the Institute of Medicine Guidelines.

Outcome	Underweight OR (95% CI)	Normal Weight OR (95% CI)	Overweight OR (95% CI)	Obese OR (95% CI)
**Gestational hypertension**	0.7[0.4–1.2]*	1.1[0.7–1.7]	0.7[0.4–1.1]*	1.0[0.7–1.5]
**Gestational diabetes**	NS^a^	1.3[1.1–1.4]*	1.2[0.9–1.4]	1.2[0.1–1.4]
**Postpartum hemorrhage**	0.6[0.4–0.9]*	0.8[0.7–0.9]*	1.1[0.8–1.4]	1.0[0.7–1.3]
**Cesarean delivery**	1.1[1.0–1.2]	0.9[0.9–1.1]	1.2[1.1–1.3]*	1.6[1.4–1.7]*
**Small for gestational age** ^**b**^	2.8[2.4–3.4]*	1.5[1.3–1.8]*	1.4[1.1–1.9]*	0.9[0.8–1.3]
**Large for gestational age** ^**c**^	0.6[0.5–0.9]*	0.7[0.7–0.8]*	1.4[1.2–1.8]*	1.5[1.2–1.8]*

^a^NS: the number in this category was too small to analyze

^b^small for gestational age and ^c^large for gestational age, defined as the < 10th percentile and > 90th percentile, respectively, of sex-specific reference curves.

Based on multiple logistic regression models adjusted for maternal age at birth, smoking, alcohol consumption, maternal height, social status defined by education, length of gestation, and residential area (urban or rural).

*Significantly different from the estimate for the reference category (p < 0.05).

### Birth outcomes according to GWG after the exclusion of cases of gestational diabetes or gestational hypertension

In this study, the OR for the delivery of an LGA infant was further analyzed based on cases without GDM, and the OR for the delivery of an SGA infant was analyzed in the cases of GWG without hypertension. We found that when the mothers with GDM were excluded, the association of excessive GWG with delivery of an LGA infant was decreased (OR 1.872; 95% CI = 1.72–2.03), whereas the relationship with delivery of an SGA infant increased steadily after accounting for the blood pressure of the low GWG group (OR 1.671; 95% CI = 1.45–1.93).

## Discussion

In this study, we comprehensively examined the independent and joint associations of pre-pregnancy BMI and GWG with maternal and neonatal outcomes in Chinese Han women. Disturbingly, we found that nearly half of the women in the local population became pregnant when their BMI was suboptimal, and in more than 60% of the pregnancies, the weight gain was not within the range recommended by the 2009 IOM guidelines. Consistent with prior research [6], we also reported that the baseline weight is one of the most significant predictive factors for weight gain in pregnancy. The women who were overweight and obese pre-pregnancy were the most likely to have a high GWG. In our study, only 10.4% of the population was overweight or obese, which is significantly lower than the prevalence in developed countries [7]. Therefore, the BMI criteria developed by the WHO are not suitable for Asian populations. However, there is no recommendation for new, clear BMI cut-off points for all Asians [8– 9]. Previously, it was reported that 73% of pregnant women have excessive GWG [10]; in contrast, the rate in our population was relatively low, which may also be due to differences in Asian versus Western populations. In general, in our population, adherence to the new guidelines was less than desirable.

Pre-pregnancy BMI was an independent predictor of pregnancy outcomes, and the contribution of GWG was relatively modest. Despite this, we concluded that excessive GWG is associated with substantial adverse feto-maternal outcomes. The mothers with excessive weight gain had a significantly higher likelihood of pregnancy-induced hypertension, postpartum hemorrhage, and cesarean delivery, and they were more likely to deliver an LGA infant than the women who gained weight within the appropriate range; these findings agree well with those of previous studies [11– 12]. Weight gain below the guidelines seemed to be slightly protective against some adverse outcomes. This finding is consistent with those of previous studies [13], although our data showed that low GWG was associated with moderate-to-strong evidence of increased risk of GDM and delivery of an SGA infant.

Similar to the findings of other researchers [14–17], we found that GWG was associated with an increased risk of pregnancy-induced hypertension and GDM. In our multivariate analysis, a J-shaped relationship was observed for the association between the risk of gestational hypertension and the categories of increasing GWG. Indeed, the risk was highest in the excessive GWG group and lowest in the inadequate GWG group. However, it is difficult to determine whether hypertension causes edema, which is refiected by greater weight gain, or whether greater weight gain causes hypertension. This finding is consistent with the findings of previous research showing that gestational hypertension is associated with an increased pre-pregnancy BMI and high GWG [6, 11]. Another interesting observation presented herein is the effect of excessive GWG on GDM. Consistent with previous research [6, 18], a tendency for an inverse relationship was observed between GWG and the risk of GDM. Because the diagnosis of GDM occurs primarily at 26–28 weeks of gestation and screening is carried out more often, treatment with diet and/or insulin plus increased physical activity may affect subsequent weight gain, resulting in decreased weight gain in late pregnancy.

To date, limited research has been conducted to examine weight gain during pregnancy and postpartum hemorrhage. Consistent with previous studies [19], we found that the women in the excessive GWG group had a higher likelihood of postpartum hemorrhage than the women in the adequate GWG group.

The cesarean section rate was high in our population, and this result is in agreement with the report by the WHO [20]. Aside from the indicated caesarean sections, the high rate of cesarean section might be influenced by socioeconomic factors and the introduction of the one-child policy in 1979. Researchers agree that there is a substantial relationship between BMI and cesarean delivery, but the relationship between maternal weight gain and cesarean delivery has recently become a controversial subject. As was the case in the present findings, some authors have proposed that the likelihood of cesarean delivery is increased in women with excessive GWG [21–22], while others have concluded that GWG does not have a significant infiuence on the occurrence of cesarean delivery [23]. Interestingly, in the current study, we also found that the likelihood of cesarean delivery was increased in women with an excessive GWG, especially in obese women, whereas the results of other studies have shown that the increase in cesarean delivery is most prominent for women with a low BMI who experienced excessive weight gain [24]. Consistent with previous studies [25], we found that the women in the excessive GWG group had a higher likelihood of emergency cesarean delivery than those in the adequate GWG group.

Research has indicated that, in addition to gestational age, pre-pregnancy weight and prenatal weight gain are also primary predictors of infant birth weight [26]. Recently, there has been increased interest in the potential consequences of excess weight gain on neonatal weight, irrespective of the woman’s size at the start of pregnancy [27]. A systematic review showed that there is strong evidence linking weight gain above the IOM recommendations with high birth weight, macrosomia and delivery of LGA infants, whereas there was only moderate evidence linking weight gain above the IOM recommendations with cesarean delivery and maternal postpartum weight retention[28]. Our principal finding relates to neonatal outcomes, and our findings particularly highlight the effects of GWG in conferring a higher risk of delivery of LGA and SGA infants. Our results confirmed that gaining less than the IOM recommendations increased the risk of delivery of an SGA infant by two-fold compared with gaining the recommended amount of weight; these data are consistent with findings from previous research[29]. As in other studies [14, 30], we also found that weight gain above the guidelines was associated with an increased risk of delivering an LGA neonate. All of these results are alarming, as they indicate the presence of a vicious cycle over generations.

Maternal BMI and GWG reflect nutritional status before and during pregnancy, respectively, and the impact of GWG should be interpreted in relation to maternal BMI. A stratified analysis showed that the effect of GWG tended to be modest across the BMI groups, but the magnitude of the association was greater for the underweight women with low GWG and SGA infants and for the obese women with high GWG and LGA infants. For many years, clinical recommendations have focused mainly on the limitation of low birth weight. However, the nutritional and clinical context has changed, and an increasing number of mothers are beginning their pregnancy overweight or obese; therefore, the risk of delivering an LGA infant must be considered. In a previous study, an increased risk of high birth weight was found with increasing maternal weight gain; however, this was not the case in underweight women [31]. In our study, the incidence of delivery of an LGA infant increased in all of the BMI groups. Moreover, M. Cedergren [32] found that the risk of cesarean delivery was increased in all maternal BMI categories; however, we found that excessive weight gain only had an effect on cesarean delivery among the overweight and obese women. This discrepancy may be due to the fact that M. Cedergren referred to weight gain < 8 kg as low weight gain and weight gain > 16 kg as high weight gain, which does not comply with the IOM recommendation. Even studies of women with normal pregnancy BMIs indicate that women who gain weight beyond the recommended IOM guidelines have an increased risk of adverse outcomes. For obese women, a GWG greater than the IOM recommendation is associated with an increased risk of delivery of an LGA infant [33], and our data did not show an increased risk of delivering an SGA infant in those with a GWG less than that recommended by the IOM.

GDM and pregnancy-induced hypertension are frequently encountered and can be important confounders for associations with birth weight, such as delivery of LGA infants in mothers with GDM or delivery of SGA infants in mothers with gestational hypertension [34–35]. Few studies have taken into account the effect of blood pressure and glucose tolerance disorders on the association between GWG and extreme birth weight. In the present study, we observed a positive association between GWG and delivery of LGA infants overall, but it decreased appreciably after adjustment for maternal GDM, which suggests that the maternal glucose concentration might be involved in the risk of delivery of LGA infants; these data conform well with those of other authors, who have shown similar effects of excessive GWG on birth weight in non-diabetic pregnant women[36]. Compared with the uncorrected data, the removal of the data obtained from women with gestational hypertension resulted in a substantial increase in the incidence of delivery of an SGA infant in the inadequate GWG group.

Overall, our study was a robust, nested cohort analysis performed retrospectively using a validated database. This study has both strengths and limitations. Some of the strengths include its multicenter design, which was nationally representative, its large sample size and the rigorous collection of data, which permitted an informative validity assessment of risk factors in the overall dataset. Because multiple births, preterm delivery and gestational age are strongly linked to weight gain, only singleton, full-term (at least 37 weeks gestation) births were included in the analysis. In contrast to the majority of previous studies that categorized weight gain by the number of weeks between measurements to obtain a weekly GWG [37], we used total GWG as our measure of exposure because our subjects were limited to full-term pregnant women only. Thus, if the most severe cases presented as preterm deliveries, they were excluded from our analyses. Using the weight and height recorded at the first prenatal visit within the first 12 weeks of pregnancy is one of the limitations of this study. Because it is not possible or practical for every woman to accurately know how much she weighs immediately before pregnancy, this study is inevitably prone to some misclassification error, which could lead to overestimation or underestimation of GWG; we acknowledge this limitation. However, currently, this is a widely used method, and it has been found to correlate well with measured pre-pregnancy weight [38–40]. Another limitation of the study is that the guidelines for GWG and BMI that we used were developed for a non-Chinese population and might not be suitable for our population, resulting in deviation. However, there is no recommendation for clear BMI or GWG cut-off points for all Asians. Additional high-quality randomized controlled trials are needed to develop ethnicity-specific guidelines for pre-pregnancy BMI and GWG for Chinese women. Studies have concluded that GWG is the strongest predictor of weight retention [41], and pregnancy might substantially increase postpartum body weight for subgroups such as those with high GWG [42]. Due to the retrospective nature of the data, some of these important pregnancy outcomes could not be assessed. This study benefited from a large sample size. It was limited, however, by the performance of joint analysis of the effects of maternal pre-pregnancy BMI and GWG on pregnancy outcomes. Because some of the subgroups were relatively small, important differences might not have been detected due to the lack of sufficient statistical power in individual categories, such as in the analysis of the relationship between GDM in underweight women and inadequate GWG. Despite these limitations, the results of this study have important clinical and public health implications.

## Conclusions

The present data suggest that the adherence of Chinese individuals to the new guidelines is less than desirable. Pregnancy is widely viewed as a period when women are open to lifestyle changes to optimize their health. Appropriate control of GWG, within the limits set by the IOM guidelines, will avoid potential neonatal complications related to birthweight and will also improve pregnancy outcomes.

## Supporting Information

S1 Table Demographic characteristics of the study population according to the maternal pre-pregnancy BMI and GWG categories. (PDF)
