# Supplementary material for: Joint and Independent Associations of Gestational Weight Gain and Pre-Pregnancy Body Mass Index with Outcomes of Pregnancy in Chinese Women: A Retrospective Cohort Study
Source: PLoS One. 2015 Aug 27;10(8):e0136850. doi: 10.1371/journal.pone.0136850 (PMC4552294; doi:10.1371/journal.pone.0136850)
Supplement: S1 Table — (PDF) [file pone.0136850.s001.pdf]

S1 Table. Demographic characteristics of the study population according to the maternal pre-pregnancy BMI[a] and GWG[b] categories.

Pre-pregnancy body mass index (kg/m^2)					*p*	institute of Medicine categories			*p*
	<18.5	18.5-24.9	25-29.9	≥30		Inadequate	Adequate	Excessive	
No. of subjects	6424	37359	4497	587		12223	17978	18666	
Age(years)	26.6±4.2	27.4±4.5	28.4±4.5	28.4±4.7	<0.001	27.2±4.67*	27.3±4.58	27.7±4.38*	<0.001
Height(cm)	161.97±4.9	161.54±4.7	161.62±4.9	161.45±5.1	0.395	160.89±4.84*	161.5±4.54	162.34±4.62*	<0.001
Maternal BMI(kg/m^2)	17.5±8.04	21.3±1.67	26.7±1.28	32.1±2.31	<0.001	21.81±2.84*	21.14±2.8	21.78±3.20*	<0.001
Gestational weight gain(kg)	15.47±4.97	14.51±4.89	12.95±5.25	12.69±6.12	<0.001	8.92±1.90*	13.4±1.94	19.14±3.97*	<0.001
Gestational age at delivery(wk)	39.5±1.5*	39.6±1.6	39.6±1.7	39.4±1.6*	<0.001	39.71±1.69*	39.65±1.6	39.52±1.52*	<0.001
Education(%)					<0.001				<0.001
University and above	3334(51.90)	19620(52.52	2391(53.17)	303(51.62)		6017(49.23)	9462(52.63)	10156(54.41)	
Junior college	1819(28.32)	10937(29.28	1426(31.71)	194(33.05)		3422(27.99)	5270(29.31)	5680(30.43)	
High school	1152(17.93)	6008(16.08)	550(12.23)	69(11.75)		2483(20.31)	2845(15.82)	2446(13.10)	
Illiteracy	9(0.14)	84(0.22)	11(0.24)	1(1.07)		301(2.46)	401(2.23)	384(2.06)	
Smoking during pregnancy,%	0.37%	0.07%	0.27%	0.51%	<0.001	0.245%	0.16%	0.46%	<0.001
Drinks alcohol(%)	1.71%	1.33%	1.38%	1.7%	<0.001	0.96%	1.2%	2.05%	<0.001
Residential areas					<0.001				<0.001
Urban	4287(66.7)	24531(65.7)	3283(73)	474(80.7)		7596(62.15)	11575(64.38)	13386(71.71)	
Rural	2137(33.3)	12828(34.3)	1214(30)	113(19.3)		4627(37.85)	6390(35.62)	5270(28.29)	

[a]BMI: body mass index； [b]GWG: gestational weight gain
